# Anticarcinogenic effects of miR-199a-loaded gold nanoparticles on hepatocellular carcinoma: in vitro study

**DOI:** 10.1038/s41598-026-42604-x

**Published:** 2026-04-02

**Authors:** Samar El Achy, Maisa E. Moustafa, Mohamed Fouad, Ashraf Awad, Reham Abdelhaleem, Thanaa Shalaby

**Affiliations:** 1 Department of Pathology, Faculty of Medicine, Alexandria University, Alexandria, 21521 Egypt; 2 Center of Excellence for Research in Regenerative Medicine and Applications (CERRMA), Faculty of Medicine, Alexandria University, Alexandria, 21521 Egypt; 3 Department of Medical Biophysics, Medical Research Institute, Alexandria University, Alexandria, 21521 Egypt; 4 Department of Biochemistry, Medical Research Institute, Alexandria University, Alexandria, 21521 Egypt; 5 Department of Clinical Pathology, Faculty of Medicine, Alexandria University, Alexandria, 21521 Egypt; 6 Nanotechnology Training Center, Medical Technology Center, Alexandria University, Alexandria, Egypt

**Keywords:** Apoptosis, Gold nanoparticles, Hepatocellular carcinoma cell line, miRNA-199a, miRNA delivery, Nano-complex, Proliferation, Biotechnology, Cancer, Drug discovery, Nanoscience and technology

## Abstract

Hepatocellular carcinoma (HCC) represents a critical oncological challenge demanding innovative therapeutic interventions. miRNA has been known to play an important role in cancer inhibition to control HCC’s development and progression by regulating cell proliferation and apoptosis. The major hurdle is to deliver the miRNA at the site of tumor. Metallic nanoparticles with modified surface can be used to solve this problem. In the current study, gold-nanoparticles (Au NPs) were prepared, and their surface was modified with PEG moiety to facilitate the attachment of miRNA. For the first time, the modified gold NPs were loaded with miR-199a. Our findings revealed that, when cells treated with gold bare (80 nM) for 24 h, a low cytotoxicity was obtained (11.11 ± 2.25%). When cells treated with nanocomplex miRNA- PEG –Au NPs (80 nM) for 24 h, a significantly increased cellular cytotoxicity was obtained (55.7 ± 4.55%). Also, the prepared nanocomplex exhibits a promising potential in suppressing tumor cell proliferation and significantly enhancing apoptosis in a concentration and time dependent manner. These results underscore the transformative potential of targeted nanomaterial-based miRNA delivery as a sophisticated therapeutic modality in cancer management. In conclusion, Au NPs are excellent carriers for miRNA where they increase the cellular uptake, exerting a promising anticancer effect on HCC cells, representing a new approach in developing precision therapeutics for hepatocellular carcinoma.

## Introduction

Hepatocellular carcinoma (HCC) is the sixth most common cancer in adults worldwide and has been reported as the most common malignancy affecting Egyptian males in 2022 (estimated incidence 24% among males)^1^. It ranks at fourth place according to cancer-related deaths worldwide and has been increasing over the last 30 years^2^. HCC is related to a number of genetic and environmental factors including cirrhosis, hepatitis C virus (HCV), Hepatitis B virus (HBV) infection and alcoholic liver damage^3^. HCC is usually diagnosed at late stages, making treatment options particularly difficult in the majority of cases. If diagnosis is made at an early stage, surgical resection, liver transplantation and radiofrequency ablation are potentially curative; whilst poor prognosis is expected in advanced cases where conventional chemotherapy has no satisfactory effect^3^. Despite improvements in treatment options, the 5-year survival rates remain dismal 20% worldwide^4^.

MicroRNAs (miRNAs) are small single-stranded, non-coding RNA molecules that modulate the translation and stability of mRNAs at the post-transcriptional level^5,6^. In HCC, miRNA expression has been linked to the pathogenesis, progression, and metastatic ability of cancer cells^7^. Additionally, different stages of HCC carcinogenesis are characterized by a distinct group of miRNAs^7^. Recent research has shown that abnormal miRNA production is linked to changes in how HCC cells move^8^. Some miRNAs have promoted HCC cell proliferation and apoptosis whilst others have been repressive^9^. These miRNAs can represent potential therapeutic targets allowing proper control over HCC’s development and progression^10^.

The miR-199 family, especially miR199a-3p and miR-199a-5p, shows consistent downregulation in HCC^11^. This occurred in over two-thirds of tumor tissues compared to non-neoplastic liver tissue, with validation by qRT-PCR and TCGA data^12–15^. Loss of miR-199a releases oncogenic targets like discoidin domain receptor-1 (DDR1), CD151, mTOR and c-Met. These changes boost cell growth, invasion, migration, and epithelial-mesenchymal transition during HCC progression^12,14,16^.

Low miR-199a levels, for example, have been reported to raise DDR1 levels to drive invasion and CD151/c-Met to aid tumour spread and growth; restoring miR-199a triggers cell death and stops cell cycle progression via matrix metalloproteinase-9 (MMP-9) and E2F3^12,14^. Low levels of miR-199a have been linked to advanced HCC stages and worse overall survival in HCC patients^13,14,16^. This “miR-199a downregulation → target activation → HCC initiation/progression” axis provides mechanistic rationale for miR-199a restoration via advanced carriers like gold nanoparticles (AuNPs) to suppress oncogenic cascades^16^.

In HCC cell lines, miR-199a was observed to inhibit tumor proliferation and promote apoptosis and cell cycle arrest through the regulation of MMP-9 expression^17^. Other identified targets of miR-199a-3p include the mammalian target of rapamycin, which are significant for the biological function of miR-199a-3p as a tumor suppressor^8^. miR-199a-5p directly inhibits the expression of DDR1, and the absence of miR-199a-5p results in the upregulation of DDR1, thereby enhancing the invasion of HCC cells^8^. A separate study found that upregulated miR-199a-5p directly inhibited E2F3 activity and increased the sensitivity of HCC cells to standard chemotherapy^18^. MiR-199a-5p markedly augmented the inhibitory effects of cisplatin on cell proliferation^19^. Cisplatin reduced miR-199a-5p levels, subsequently inducing autophagy through the activation of autophagy-associated gene 7, which is a direct target of miR-199a-5p^20^.

The therapeutic application of miRNAs in cancer treatment, faces significant hurdles due to their inherent instability, poor cellular uptake and rapid degradation in biological fluids^21,22^. Traditional delivery methods, such as viral vectors and cationic liposomes, often present substantial limitations including immunogenicity, cytotoxicity, and low transfection efficiencies^23^. For instance, cationic lipid formulations, while widely explored, typically exhibit dose-dependent cytotoxicity with reported half maximal inhibitory concentrations (IC50) often below 10 µM in some cell lines, alongside transfection efficiencies that frequently remain below 70% in vitro.^24^ Similarly polymeric nanoparticles, while offering some improvement in biocompatibility, can suffer from off-target effects and inconsistent miRNA release kinetics^23^. These shortcomings underscore the critical need for novel, more effective delivery systems that can enhance therapeutic efficiency while minimizing adverse effects.

Among the inorganic nanoparticles, gold nanoparticles (AuNPs) emerge as particularly promising candidates, due to their biocompatibility, large surface area, and facile surface functionalization, which enable high-affinity biomolecule conjugation^25,26^. AuNPs are commonly employed for the delivery of miRNA mimics; however, their application is limited by the complex procedures required for the conjugation of miRNA to the AuNPs. Thiol and amino functional groups can be readily conjugated to the surface of AuNPs, and these chemically modified AuNPs have been utilized as carriers for miRNA^27^. Jia et al. documented the covalent conjugation of thiol-modified antagomir-miRNA-155 to gold nanoparticles (Au NPs) and the administration of miRNA-155-AuNP^28^. Additionally, AuNPs offer higher nucleic acid carrying capacity, superior biocompatibility, low toxicity (no effects up to 187.5ug in MTT assays), rapid cellular internalization and functional delivery achieving significant miR knockdown^28,29^. To the best of our knowledge, no study has been published yet on the fabrication of nanocomlex for the delivery of miR-199a to control the growth of HCC. This study presents a novel simplified method for fabricating miRNA-loaded gold nanoparticles for the delivery of miR-199a. This process has the potential to establish PEGylated AuNP as a universal carrier for miRNA mimic. The aim of the present study was to prepare and characterize miRNA-199a loaded AuNPs, to validate the superiority of PEGylated AuNPs as a delivery vehicle for miR-199a, and to assess its effect on HCC cell dynamics including proliferation as well as apoptosis in vitro cultured HCC cell line.

## Results and discussion

### Synthesis of PEG capped Au NPs

Au NPs are recognized as promising candidates due to their straightforward synthesis, high biocompatibility with cells and tissues, ease of surface modification and conjugation, and capacity for cellular penetration. The absence of cytotoxicity in Au NPs may be beneficial for their application as delivery carriers for nucleic acids. The citrate capping method was used to reduce their toxicity^30^. All experiments utilized DEPC-treated Au NPs to avoid the presence of RNAases that could potentially degrade the miRNA. Reports indicate that DEPC-treated Au NPs exhibit no differences in their physicochemical properties compared to untreated Au NPs^31^. DEPC-treated Au NPs were initially allowed to bind with NH2-PEG-SH, followed by binding with miRNA. The loading of miRNA onto Au NPs occurs through a two-step process. The initial step involves the attachment of NH2-PEG-SH, which enhances the stability and biocompatibility of Au NPs while imparting a positive zeta potential that facilitates miRNA binding. NH2-PEG-SH capped Au nanoparticles were incubated with miRNA199a to facilitate attachment, after which the excess NH2-PEG-SH was eliminated. To our knowledge, it is the first time to use functionalized gold nanoparticles to carry miRNA199a to treat hepatocellular carcinoma in vitro. In this study, we developed a simpler method for the fabrication of miRNA loaded gold nanoparticles (nano-complex) to deliver miR-199a into the hepatic cancer cells HepG2 cells. To the best of our knowledge, no study has been published yet on the preparation of Au-PEG-miRNA199a for treatment of hepatocellular carcinoma in vitro. Our results revealed that, miR-199a effectively entered into the cytoplasm of hepatic cells in vitro assisted by the PEGylated Au NPs, exhibiting a high biological activity at low doses, indicating that Au NPs offers significant advantages over other gene delivery vectors.

### Characterization of prepared Au NPs and Au-mi-RNA 199a

#### Transmission electron microscope (TEM)

**Fig. 1:**
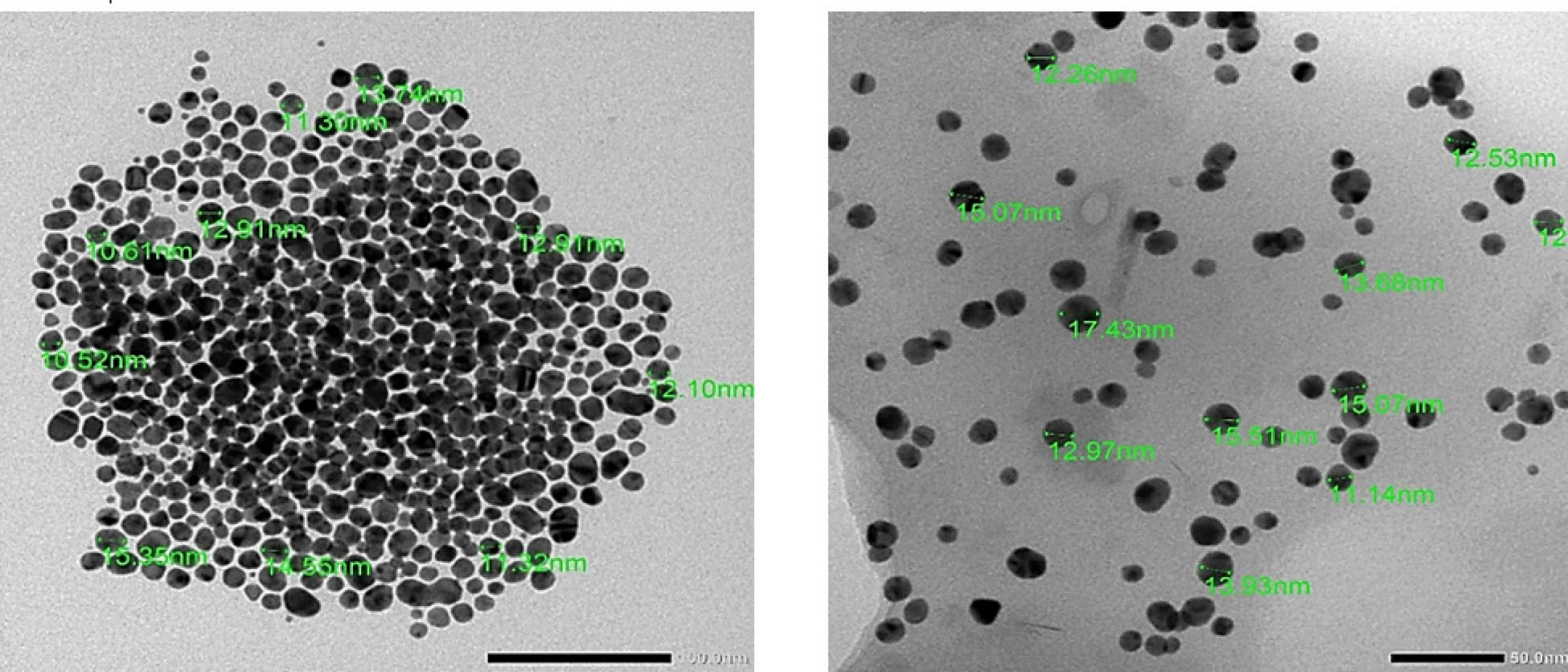
Transmission Electron Microscopy (TEM) image obtained for Au NPs (left), and miRNA-PEG-Au NPs (right).

The size and shape of PEG– Au NPs, and miRNA-PEG-AuNPs were determined by TEM. PEG– Au NPs were spherical shape with very good dispersion and an average size of 10.52–15.3 nm (Fig. 1). This size was increased after loading miRNA to 12.2–17.43 nm which is very good size for maximums the uptake efficiency^32^. The increase in size indicated the attachment of the mi-RNA to the surface of the gold.

#### Particle size analysis and zeta potential measurements

The average hydrodynamic diameter of the Au NPs, Au-PEG NPs and Au–PEG–miRNA as measured by dynamic light scattering (DLS) was 15 nm, 18.3 nm and 31.2 nm respectively. The hydrodynamic size increased from 15 to 18.3 nm, confirming PEG molecule binding to Au NPs. Furthermore, attaching PEG to Au nanoparticles causes a zeta potential change from − 27 mV to + 13.5 mV, since NH2- PEG-SH carries a positive charge. When positively charged PEG-Au NPs interact with negatively charged miRNA199a, their hydrodynamic size increases to 31.2 nm and their zeta potential drops to -19.2 mV (Table 1). Positively charged Au NPs may form polymeric complexes with negatively charged therapeutic agents like miRNA, siRNA, etc. via electrostatic interactions between the miRNA phosphate group and PEG-Au NPs’ amine group^33^. DLS data yielded poly dispersity index (PDI), which measures particle population size uniformity. PDI of the Au NPs, PEG coated gold nanoparticles and Au–PEG–MiRNA were 0.235, 0.132 and 0.152 respectively.

**Table 1:** Hydrodynamic size and zeta potential using dynamic light scattering:

	Hydrodynamic size (d nm)	PDI	Zeta potential(mV)
Au NPs	15 ± 1.25 nm	0.235 ± 0.02	-27 ± 2.15 mV
Au–PEG	18.3 ± 2.15 nm	0.132 ± 0.01	13.5 ± 1.75 mV
Au–PEG–MiRNA	31.2 ± 1.99 nm	0.152 ± 0.03	-19.2 ± 2.05 mV

#### The UV–Vis spectral analysis

**Fig. 2:**
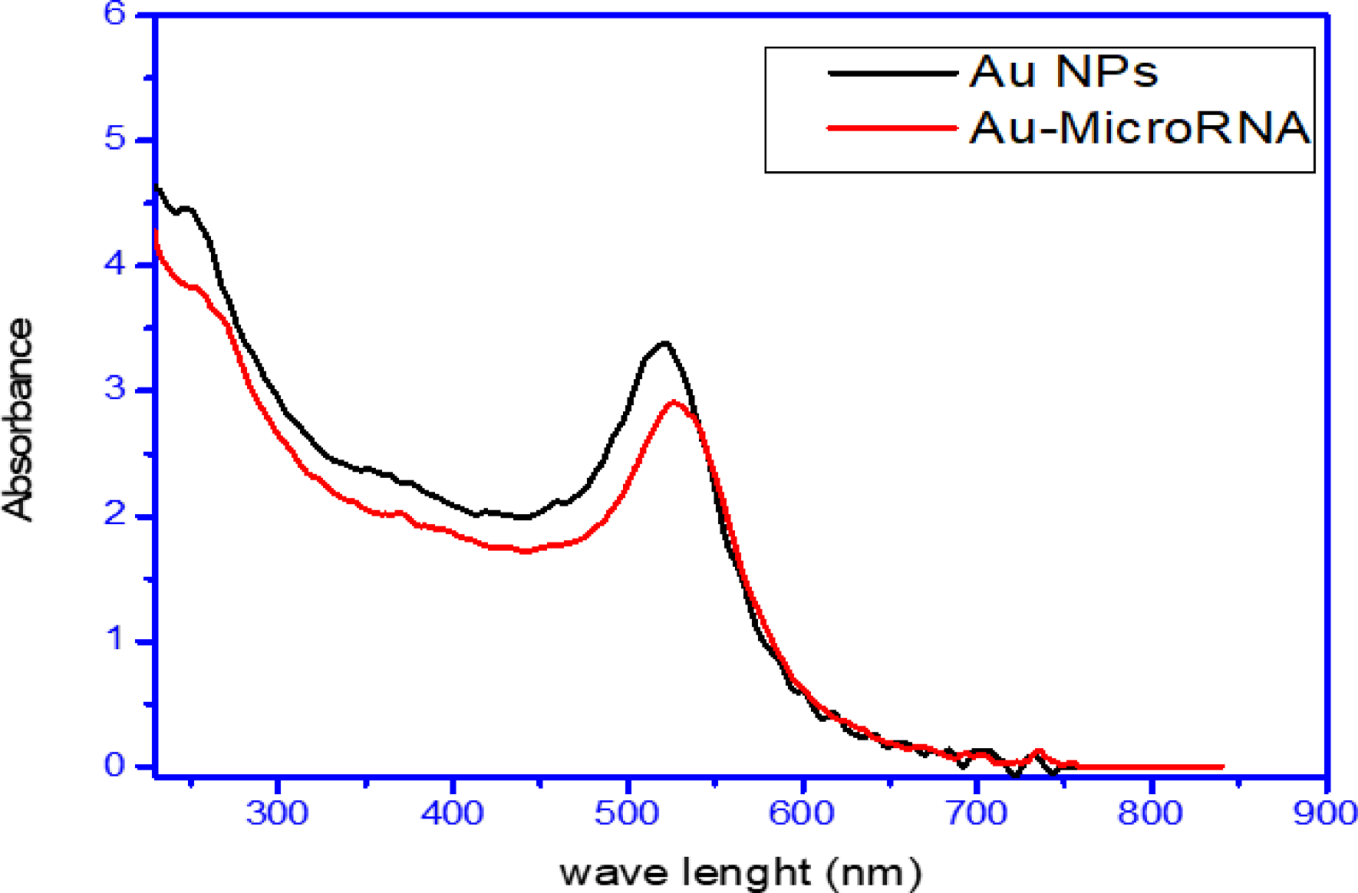
UV-Vis spectra for gold nanoparticles and miRNA–PEG–Au NPs.

UV–vis absorption spectroscopy is the most common approach for measuring nanoparticle optical and electrical characteristics because absorption bands are linked to metal nanoparticle diameter and aspect ratio. UV/vis absorption spectroscopy assessed Au-PEG NPs and Au–PEG–miRNA optical characteristics. It is widely known that surface plasma resonances cause ultraviolet and visible absorption bands in metal colloidal dispersions. Surface Plasmon oscillations are resonant. The UV–Vis spectral study of PEG–AuNPs and miRNA–PEG–AuNPs indicated a 10 nm change in the plasmonic resonance peak location. (Fig. 2). Particularly, PEG – AuNPs exhibit a surface Plasmon peak at 521 nm and miRNA–PEG–Au NPs exhibit a surface Plasmon peak at 531 nm. This change may be related to the binding of NH2- PEG-SH to Au NPs and miRNA attachment, which increases nanoparticle size^34^ .

#### PEG-Au NPs- miRNA loading efficiency

PEG-capped Au NPs were agitated with 1000 nM miRNA for 24. After incubation, nanoparticles were centrifuged and spectrophotometers assessed unbound miRNA in the supernatant. PEG-capped Au NPs loaded miRNA 75% efficiently. After incubation of the nanocomplex for two weeks in serum, the loading efficiency was 74%. This indicated the stability of the prepared nanocomplex miRNA–PEG–Au NPs.

#### Cytotoxicity of miRNA–PEG–AuNPs

**Fig. 3:**
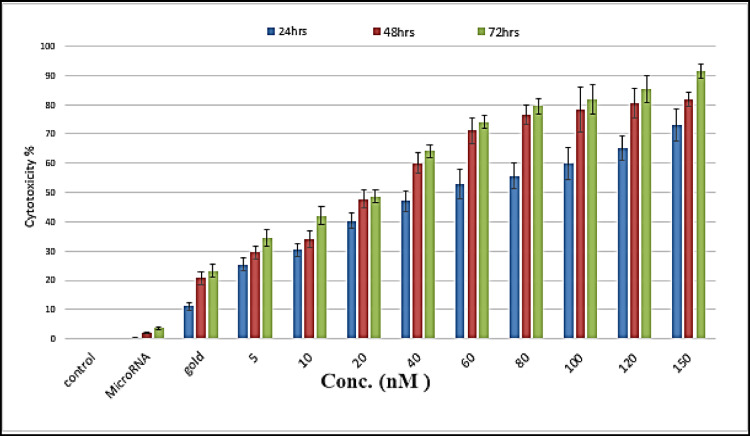
MiRNA-coated Au NPs’ fractional MTT decreased with concentration and time in HepG2 cells as compared with untreated cells (negative control) and cells treated with mi- RNA 199a (80 nM) and AuNPs (80 nM) (positive control). Values represent mean ± S.E. of three independent experiments.

**Fig. 4:**
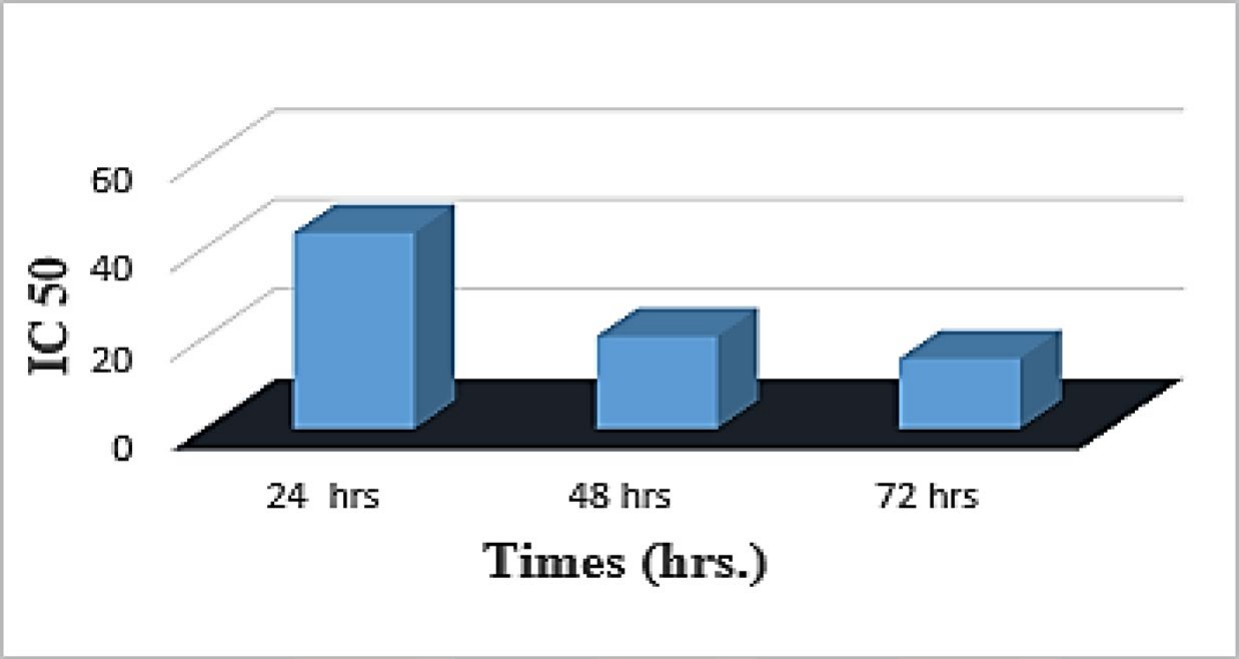
IC50 at different time points.

Cytotoxicity of the different formulations was assessed on HepG2 cell lines. Cell viability percentages were determined through the 3-(4,5-dimethylthiazol-2-yl)-2,5-diphenyltetrazolium bromide (MTT) assay relative to the control groups (Fig. 3). The percentage of viable cells (%viability) after treatment of cells with various concentration of miRNA–PEG–AuNPs was determined and subsequently the half maximal inhibitory concentrations (IC_50_) was calculated. The effect of bare AuNPs was minimal compared to the conjugated nanocomplex, scoring a percentage viability of 11.11%±1.21, 20.71%±2.31, and 23.18%±2.10 at 24 h, 48 h and 72 h respectively. mi -RNA loaded PEG-AuNPs exhibited a dose and time-dependent cytotoxic effect on HepG2 cell lines. miRNA-PEG-Au NPs showed an approximately 3-fold increase in its cytotoxic effect when the concentration was increased 30 folds (from 5nM to 150nM) at the 3 time points investigated in this study (24 h, 48 h, and 72 h). Additionally, the calculated IC_50_ value decreased with longer incubation time points scoring 43.70 nM, 20.52 nM, 15.58 nM after 24 h, 48 h and 72 h respectively (Fig. 4). This behavior parallels the dose-responsive profile of AuNP-anti-miR-211 complexes, which exhibited negligible cytotoxicity (20% viability loss) in normal Huh7 hepatocytes up to 1.2 nmol/L, while potently inhibiting HepG2 proliferation via CCK-8 assays^35^. Likewise, PLGA-b-PEG-encapsulated miRNAs (e.g., antimir-21/10b) induced graded apoptotic escalation in doxorubicin-challenged HepG2 cells, underscoring nanoparticle-mediated nucleic acid delivery’s tunable potency^36^. Conversely, green-synthesized AuNPs displayed higher IC50 thresholds (10–59 ug/ml) against HepG2, likely due to disparate capping agents impairing nM-scale efficacy^37^. Relative to cytotoxic polymers like PEI, our formulation’s nM-range selectivity affirms PEG-AuNPs’ biocompatibility edge for miRNA therapeutics, as corroborated in AuNP-miRNA overviews^25^. Our data indicates that when miR199a transfected with gold nanocomplexes resulted in a higher cytotoxic efficiency represented by higher cell death, most likely through increasing apoptosis^38^. It also proves that the PEG-Au NPs is efficient and safe for delivery of fragile nucleotides with adequate concentrations for effective treatment.

#### Cytotoxicity using flow cytometry

**Fig. 5:**
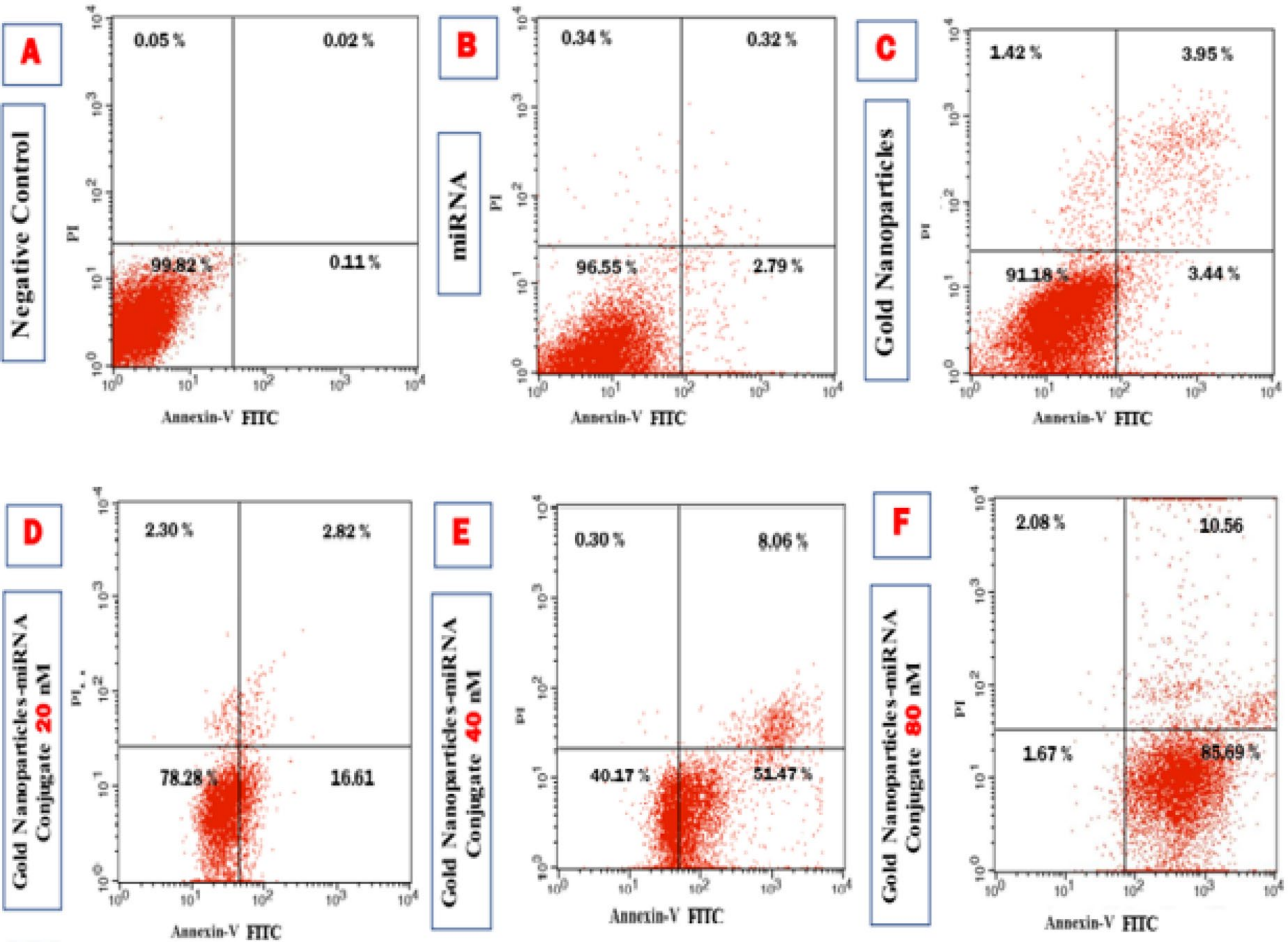
Flow cytometric analysis showing Annexin V-FITC and PI dot plot with quadrant gates of the four populations to evaluate the apoptosis in the HepG-2 cells for each of the control and studied groups.

**Fig. 6:**
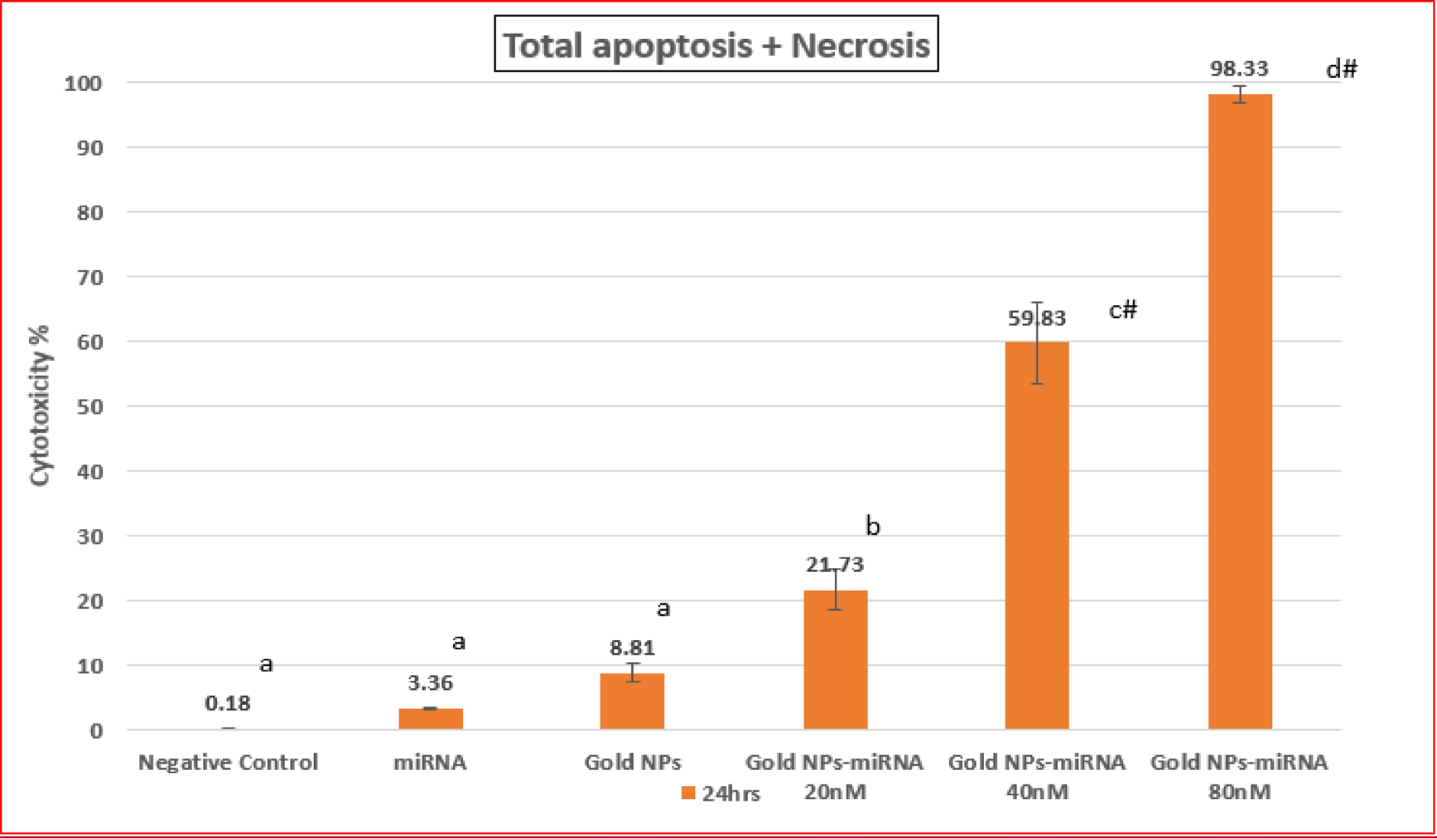
Bar chart of the Annexin V-FITC apoptosis assay results demonstrating the concentration-dependent effect of Gold NP-miRNA Conjugate on the HepG2 cells at 24-hours.

Flow cytometry with Annexin V/PI staining robustly quantifies apoptosis by detecting phosphatidylserine externalization in early apoptotic and PI uptake in late apoptotic/necrotic cells, providing a reliable distinction from viable, or necrotic cell populations^36–39^. Flow cytometry analysis using Annexin V, FITC conjugate and PI was conducted to assess apoptosis in HepG-2 cells across different experimental groups, with quadrant gates identifying four cell populations (Fig. 5). The cells were incubated for 24 h, in the untreated cells (negative control), the majority remained viable (99.82%), with a small fraction being apoptotic (0.13%) and necrotic (0.05%) (Fig. 5A). Cells exposed to miR-199a alone for 24 h showed a high viability (96.55%), with low apoptosis (3.11%), and necrosis (0.34%) (Fig. 5B), while cells treated with PEG-AuNPs alone for the same time period mildly increased apoptosis (7.39%), at viability (91.18%), and necrosis (1.42%) (Fig. 5C). miR-199a-PEG-AuNPs markedly boosted Annexin V-positive population concentrations, in a concentration dependent manner; 19.43% apoptosis at 20nM (78.28 viability, 2.30% necrosis) (Fig. 5D), 59.53% apoptosis at 40nM (40.17% viability, 0.30% necrosis) (Fig. 5E), and 96.25% apoptosis at 80nM (1.67% viability, 2.08% necrosis) (Fig. 5F), these data confirming AuNP-facilitated miRNA uptake without causing substantial necrosis. This aligns with the AuNPs-miR-221 dose-dependent decrease in HepG2 cell viability, beyond bare AuNPs, via enhanced targeting and internalization, reported by Cai et al.^35^ The predominant rate of apoptosis, over necrosis (< 2.3%) in this study, supports the miR-199a’s targeted antitumor action as its restoration induces HCC cell apoptosis as reported by Mao & Wang 2015^14^. The data exhibits the presence of early apoptotic cells after the incubation with gold nanoparticles-miRNA conjugate for 24 h (Fig. 6).

### Uptake of Au NPs-miRNA by HepG2 cell lines

#### Uptake by confocal laser microscopy

**Fig. 7:**
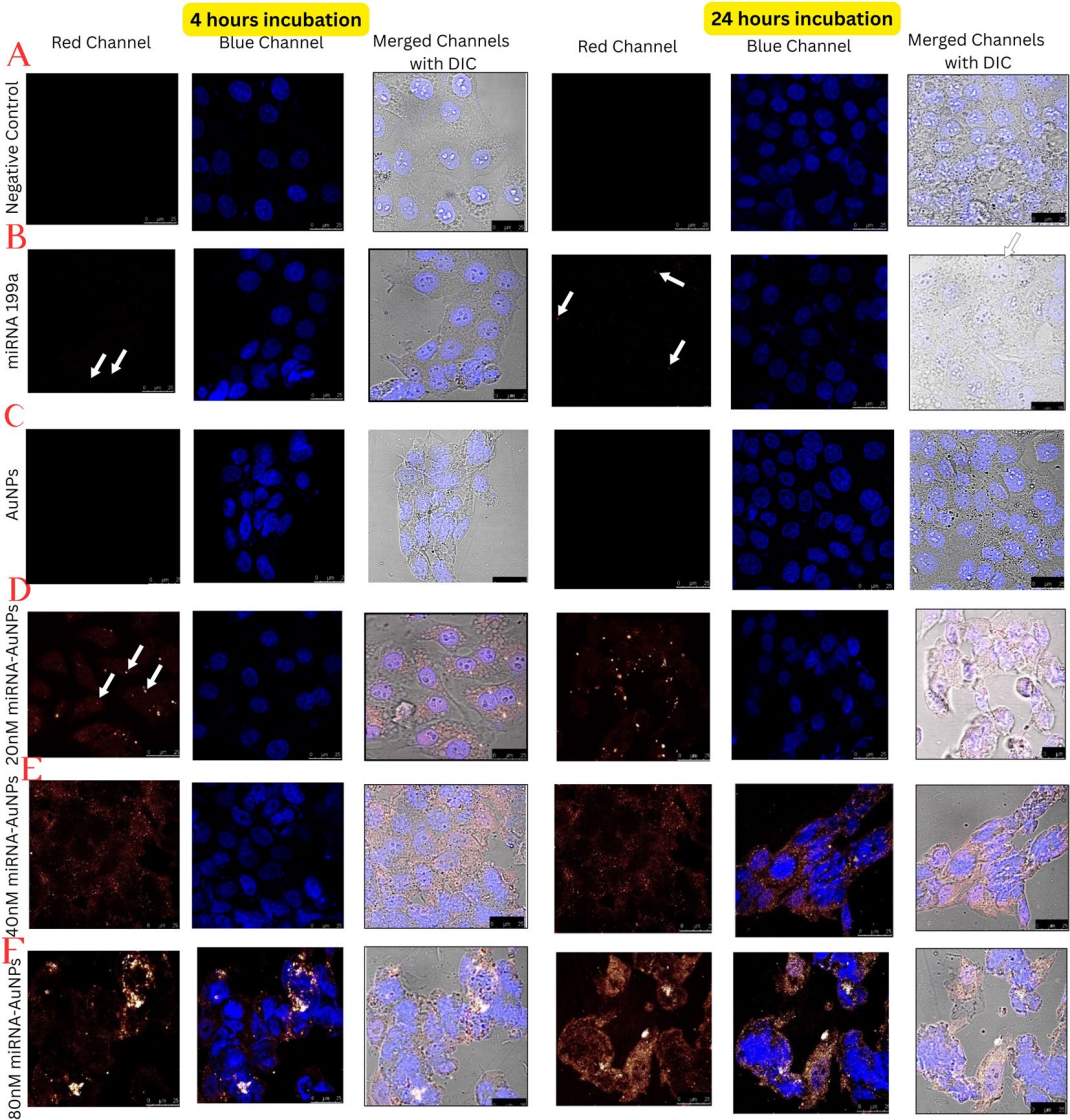
Confocal Laser Scanning Photomicrograph of negative control HepG-2 cells (first row), HepG-2 cells treated with miRNA/Cy5 (2nd row), HepG-2 cells treated with gold nanoparticles (3rd row), HepG-2 cells treated with 20 nM miRNA–PEG–AuNPs (4th row), HepG-2 cells treated with 40 nM miRNA–PEG–AuNPs (5th row), HepG-2 cells treated with 80 nM miRNA–PEG–AuNPs (6th row) after 4 and 24 h incubation time. The blue fluorescence represents the nuclear counterstaining with Hoechst stain, the negative control cells showed no red fluorescence (black background). DIC mode showing the epithelial-like morphology of the HepG2 cells in small aggregates. Merged image from blue channel and red channel.

Confocal laser microscopy was performed to confirm uptake of miRNA–PEG–AuNPs by HepG2 cells. In the present study, the fluorescent images of HepG-2 were evaluated for the detection and localization of miRNA conjugated with Cy5 using confocal microscope at two-time intervals (4 and 24 h). The red fluorescence in the cytoplasm and/or in the nuclei indicated the presence of the miRNA/Cy5. Moreover, the nuclei were counterstained with Hoechst 33,342 stained in blue. Cell morphology was assessed in both the fluorescent and differential interference contrast (DIC) phase. As shown in Fig. 7, negative control cells showed homogeneous nuclear staining indicating normal proliferation (Fig. 7A). Furthermore, both cells treated with miRNA and AuNPs demonstrated a quiet homogeneous nuclear staining when compared to the negative control group (Fig. 7B and C). On the contrary, following treatment with miRNA–PEG–AuNPs conjugated with Cy5, treated HepG2 cells exhibited different degrees of apoptotic changes including loss of cell adhesion, membrane shrinkage, membrane blebbing, cytoplasmic vacuolization, cell fragmentation, and formation of apoptotic bodies the number and severity depended on the concentration of Au-miRNA conjugates (Fig. 7D–F). This mirrors reports from Cai et al., 2019, where Cy3-labelled AuNP-anti-miR-221 exhibited strong red fluorescence signals within the HepG2 cells using confocal micrscopy, confirming AuNPs’ superior uptake over free nuceli acids^35^. Similarly, epigallocatechin gallate-capped AuNPs enhanced miR-34a/let-7a delivery in HepG2 cells, while the gold nanorods induced comparable morphological apoptotic changes^40^. At lower concentrations, the changes were less severe and more limited so that cell shrinkage and membrane blebbing comprised most changes while higher concentrations, the changes were much more severe including cytoplasmic swelling, cell fragmentation, and formation of apoptotic bodies.

**Fig. 8:**
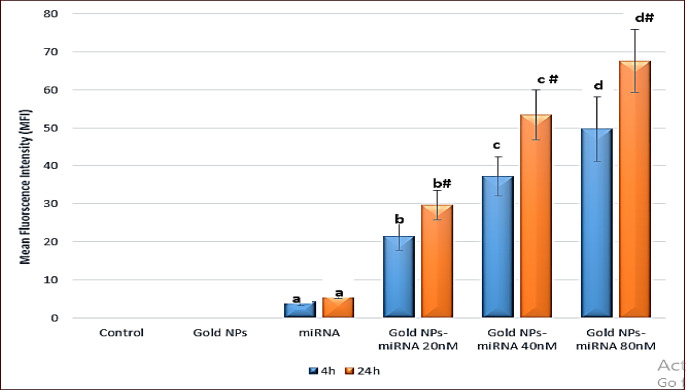
Bar chart showing the mean fluorescence intensity (MFI) in the different studied groups by confocal microscopy. P: P value for comparing the different groups. Means with common letters are not significant, while means with different letters are significant. *: Significantly significant at *p* ≤ 0.05. #: # Significantly significant within the same group at different time intervals (4 &  24 h).

Confocal microscopy revealed robust, concentration- and time-dependent cellular uptake of Cy5-labeled miR-199a-PEG-AuNPs in HepG2 cells. Both negative control and cells treated with AuNPs failed to show any red fluorescence due to absence of the fluorochrome Cy5, thus acting as a zero-fluorescence base line. Conversely, cells treated with miRNA/Cy5 showed a weak red cytoplasmic staining (Fig. 7, 2nd row) as compared to negative control group with a mean fluorescence intensity (MFI) of 4.35 ± 1.02 and 6.19 ± 1.08 at 4- and 24-hours’ time intervals, respectively. However, cells treated with miRNA–PEG–AuNPs showed red fluorescent signals of variable intensities in a concentration and incubation-time-dependent manner. Cells treated with 20 nM miRNA–PEG–AuNPs conjugated with Cy5 showed a significant MFI of 21.47 ± 3.61 and 29.75 ± 3.88 at 4- and 24-hours’ incubation, respectively when compared with negative control group. Meanwhile, in the cells treated with 40 nM miRNA–PEG–AuNPs conjugated with Cy5, the MFI significantly increased (*p* ≤ 0.05) as compared to the negative control group reaching a value of 37.28 ± 5.09 and 51.41 ± 6.57 at 4- and 24-hours’ incubation, respectively. The highest MFI in the HepG-2 cells was observed in the 80 nM miRNA–PEG–AuNPs conjugated with Cy5 treated cells resulting in 49.63 ± 8.51 & 67.59 ± 8.32 mean values after 4- and 24-hours’ incubation, respectively, which were significantly higher (*p* ≤ 0.05) than that of all the studied groups. These results indicated that Au NPs facilitate the transfection of miRNA. In alignment with our results, Baghani et al., 2022 showed an efficient cellular uptake of ssRNA-AuNPs in MCF-7 breast cancer cell lines^41^. In the present study, PEG-AuNPs exhibited a sharper dose-dependent and time-dependent kinetics, as regards to cellular uptake, which underscores the role of PEGylation in HCC-specific endocytosis, this mirrors the findings of Pourali et al., 2024, who investigated different capping agents for AuNPs^29^. The mean fluorescence intensity (MFI) in the different studied groups by confocal microscopy was represented in (Fig. 8).

#### Cellular uptake using transmission electron microscope

**Fig. 9:**
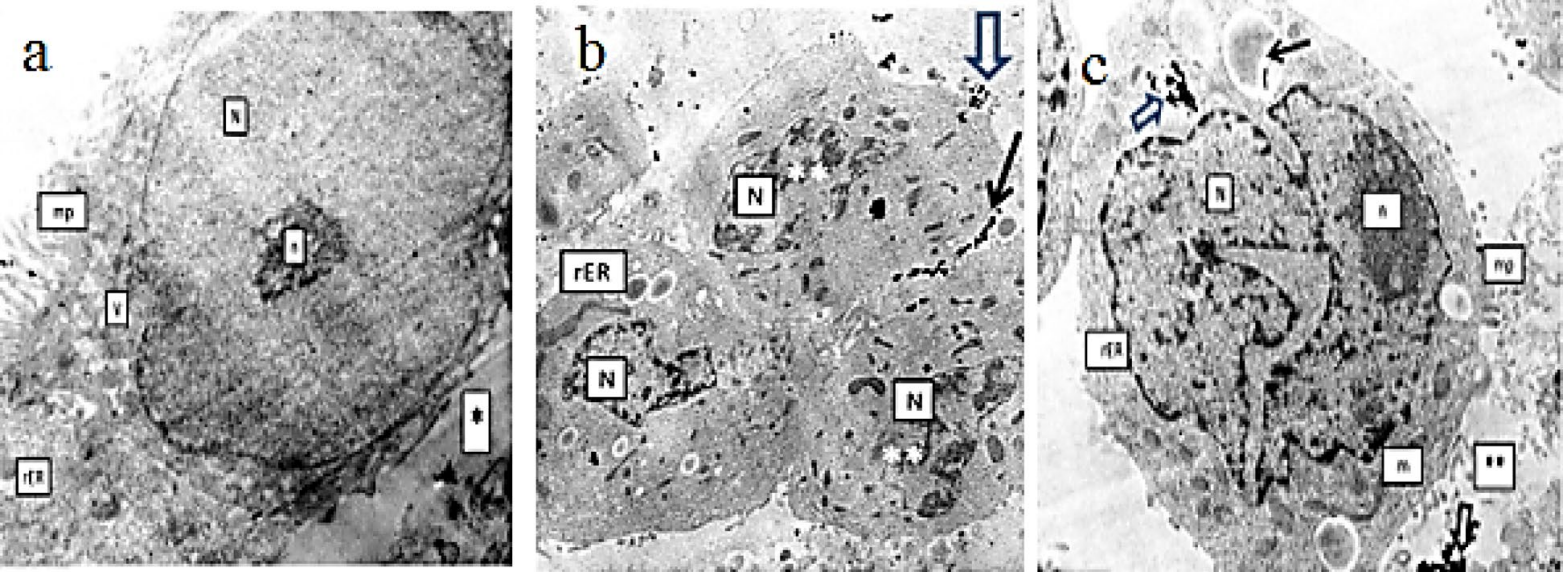
TE photomicrograph of (**a**) untreated HepG2 cells showing a large euchromatic nucleus (N) with a prominent nucleolus (n). The cell membrane exhibits irregular microplica (mp) at the free border of the cell but less prominent surface projections towards the intercellular space (*). rough endoplasm (rER) with dilated cistera, Mag. x 10,000. (**b**) Incubation of HepG2 cells with miRNA–PEG–AuNPs for 4 h, note the aggregation of dense black AuNp on the outer surface of one cell (black arrow) as well as inside the microplica (mp) of its free border (hollow arrow), Mag. x 6000. (**c**) HepG2 cell after 24 h incubation with miRNA–PEG–AuNPs revealing uptake of the NPs inside an endocytotic vesicle (hollow arrow) while other are still in the extracellular space (**), Mag. x 10,000.

Cell uptake studies clearly confirmed that miR-199a effectively entered into the cytoplasm assisted by the Au NPs, exhibiting high biological activity at low doses, indicating that Au NPs offers significant advantages over other gene delivery vectors. TEM images (Fig. 9) could confirm that NPs were localized in cytoplasmic vesicles of different sizes, which indicates endocytic NPs uptake.

The untreated HepG2 demonstrated typical ultrastructural features for cancer cells, namely large euchromatic nuclei with prominent nucleoli and irregular nuclear membranes, well developed rough endoplasm, filled with moderately dense protein products and irregular surface microplica (Fig. 9a). The latter are used by the cancer cells for nutrient uptake from the extracellular space. After 4-hour incubation period with miRNA-PEG-AuNPs (concentration of 80nM) (Fig. 9b), the HepG2 cells demonstrated aggregation of the NPs on the cell surface or adherent to the membranes of the microplica. It seemed that the 4 h duration was not a time lapse sufficient for penetration of the Au NPs into the cell cytoplasm. Following 24 h, the NPs appeared as dense black granules within the endosomal compartment of the HepG2 cells. (Fig. 9c)

#### Ki-67 flow cytometry proliferation assay

**Fig. 10:**
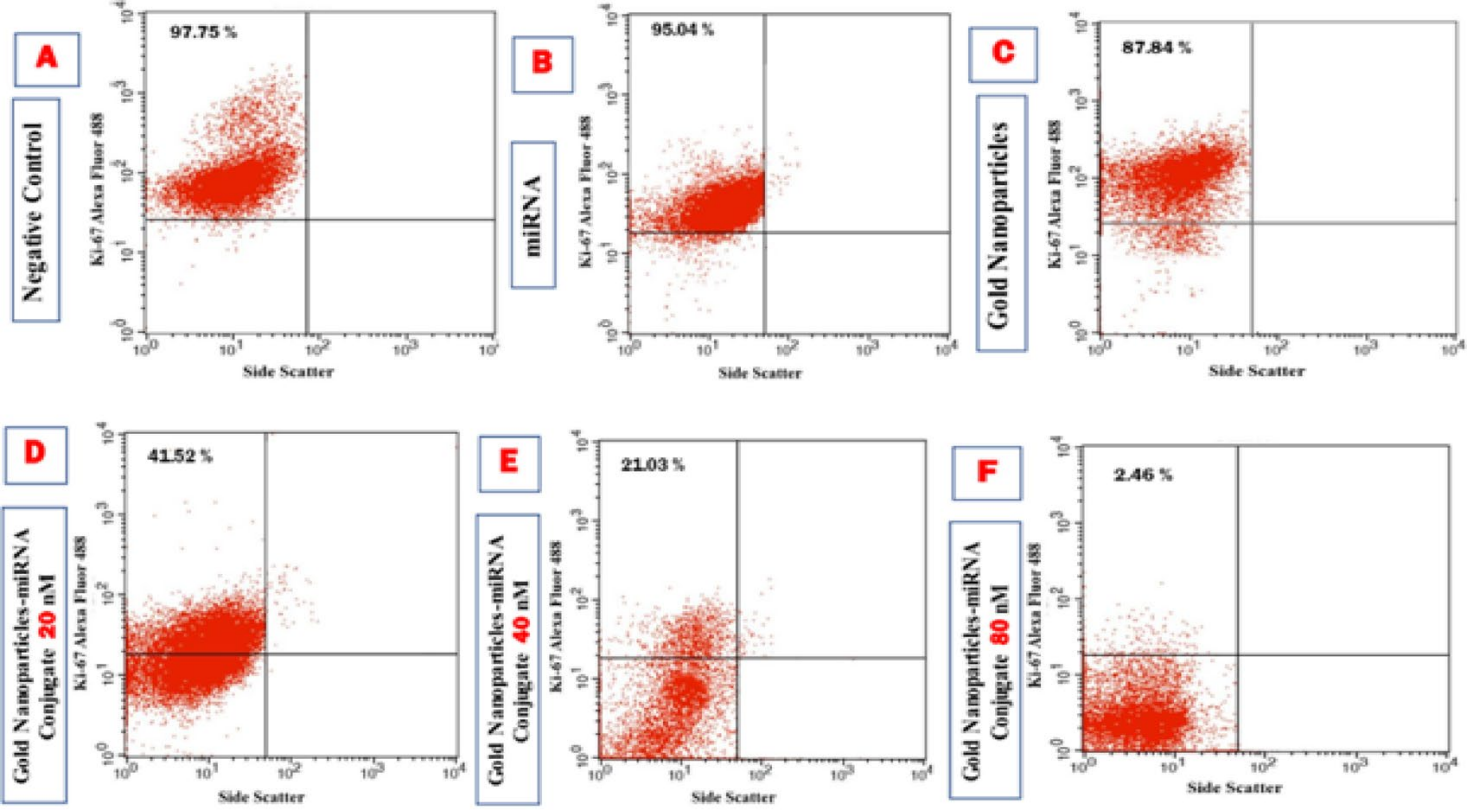
Analysis of the HepG-2 proliferation status by the Ki-67 conjugated by Alexa fluor 488 staining as shown by flow cytometry scatter plots for the different control and studied groups. (**A**) the untreated cells. (**B**) cells treated with miRNA-199a (80 nM). (**C**) cells treated with gold NPs (80 nM). (**D**) cells treated with miRNA–PEG–AuNPs (20nM). (**E**) cells treated with miRNA–PEG–AuNPs (40nM). (**F**) cells treated with miRNA–PEG–AuNPs (80nM).

To evaluate the impact of gold nanoparticles-miRNA conjugates on the proliferative capacity of HepG-2 cells, flow cytometry was used to assess the expression of the Ki-67 proliferation marker. In the flow cytometric scatterplot (Fig. 19A-F), the Upper Left (UL) quadrant represented Ki-67 positive (proliferating) cells, while the Lower Left (LL) quadrant indicated non-proliferating cells^42^. The untreated cells (Group 1) (Fig. 10A), cells treated with miRNA (Group 2) (Fig. 10B), gold nanoparticles (Group 3) (Fig. 10C), and cells treated with gold nanoparticles-miRNA conjugates at various concentrations (Groups 4a–c) (Fig. 10D–F), were analyzed for Ki-67 expression at 24 h after incubation.

As shown in (Fig. 10), all experimental groups expressed Ki-67 at varying levels. In the untreated group (negative control), the percentage of proliferating cells was 97.75 ± 1.41% at 24 h. The proliferation rates in the miRNA (Group 2) and gold nanoparticles (Group 3) groups were not significant to the control group, with proliferating cells making up 95.04 ± 3.08% and 87.84 ± 4.25%, respectively, at 24 h.

**Fig. 11:**
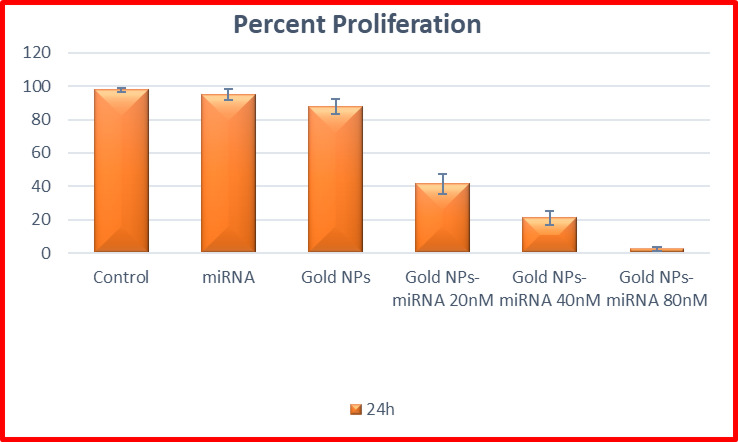
Bar chart showing the percentage of ki-67 positive cells (proliferating cells) in the different studied groups.

In contrast, cells treated with 20 nM and 40 nM gold nanoparticles-miRNA conjugates (Group 4) showed a significant decrease in proliferation, with Ki-67-positive cells at 41.52 ± 5.93% and 21.03 ± 4.29%, respectively, at 24 h. Both concentrations showed significant reductions compared to each other. The most significant decrease in cell proliferation was observed in the 80 nM gold nanoparticles-miRNA conjugate-treated cells, where only 2.46 ± 1.01% of cells were proliferating after 24 h, respectively. These values were significantly lower than those of all other experimental groups (Fig. 11). This mirrors reports by Cai et al., 2019, where AuNP-anti-miR221’s showed a potent capacity to inhibit Hep2 cell proliferation, greater than free anti-miR, via enhanced intracellular delivery^35^. Similarly, Mostafa et al., 2020, reported that epigallocatechin gallate-capped AuNPs carrying miR34a/let-7a, exhibited superior inhibition of proliferative capacity in HepG2 cells^40^. In contrast to the moderate antiproliferative effects of exosome-delivered miR-26a reported by Mahati et al., 2021^43^, in the present study the developed nanocomplex exhibited a near complete suppression of Ki67 proliferative index at miR-199a concentration of 80nM, which highlights the superior loading, uptake and inhibition of proliferation capabilities of miR-199a-PEG-AuNPs.

## Conclusion

The current study presented an efficient method for intracellular delivery of sensitive nucleotides such as miRNA. The synthesized PEGylated AuNP complexes had shown to form stable and strong electrostatic bonds with miRNA, forming an advantageous anticancer therapeutic agent. For the first time, extremely small nanomolar concentrations of miR-199a loaded onto functionalized Au NPs, forming nano-complexes capable of efficient intracellular delivery and induction of apoptosis and reduction of cell proliferation capacity in in vitro cultured HepG2 cell lines. Extremely small nanomolar concentrations of miR-199a loaded onto the Au nano-complexes were capable of efficient intracellular delivery and induction of apoptosis and reduction of cell proliferation capacity in in vitro cultured HepG2 cell lines. The key limitation in this study was its confinement to cell line models, lacking validation in physiologically relevant in vivo settings. Future investigations should prioritize orthotopic or subcutaneous tumor-bearing mouse models to assess the nano-complex’s antitumor efficacy, tumor regression, biodistribution via imaging, pharmacokinetic profiles, metabolic clearance, and long-term biocompatibility and toxicity through histopathology and serum biomarkers. Future studies should measure mRNA levels of key miR-199a targets (e.g., MMP-9, DDR1, E2F3), as well as miR-199a, using qRT-PCR and assessing their protein expression via Western blot to verify whether the nanocomplexes exert their biological effects through modulation of these genes. These experiments will bridge the translational gap, pave the way for the future development of gold nanoparticle-based delivery systems as a promising tool in precision anticancer therapies, offering enhanced targeting, reduced systemic toxicity, and the potential for personalized treatment strategies through the epigenetic modulation of specific oncogenic pathways.

**Fig. 12:**
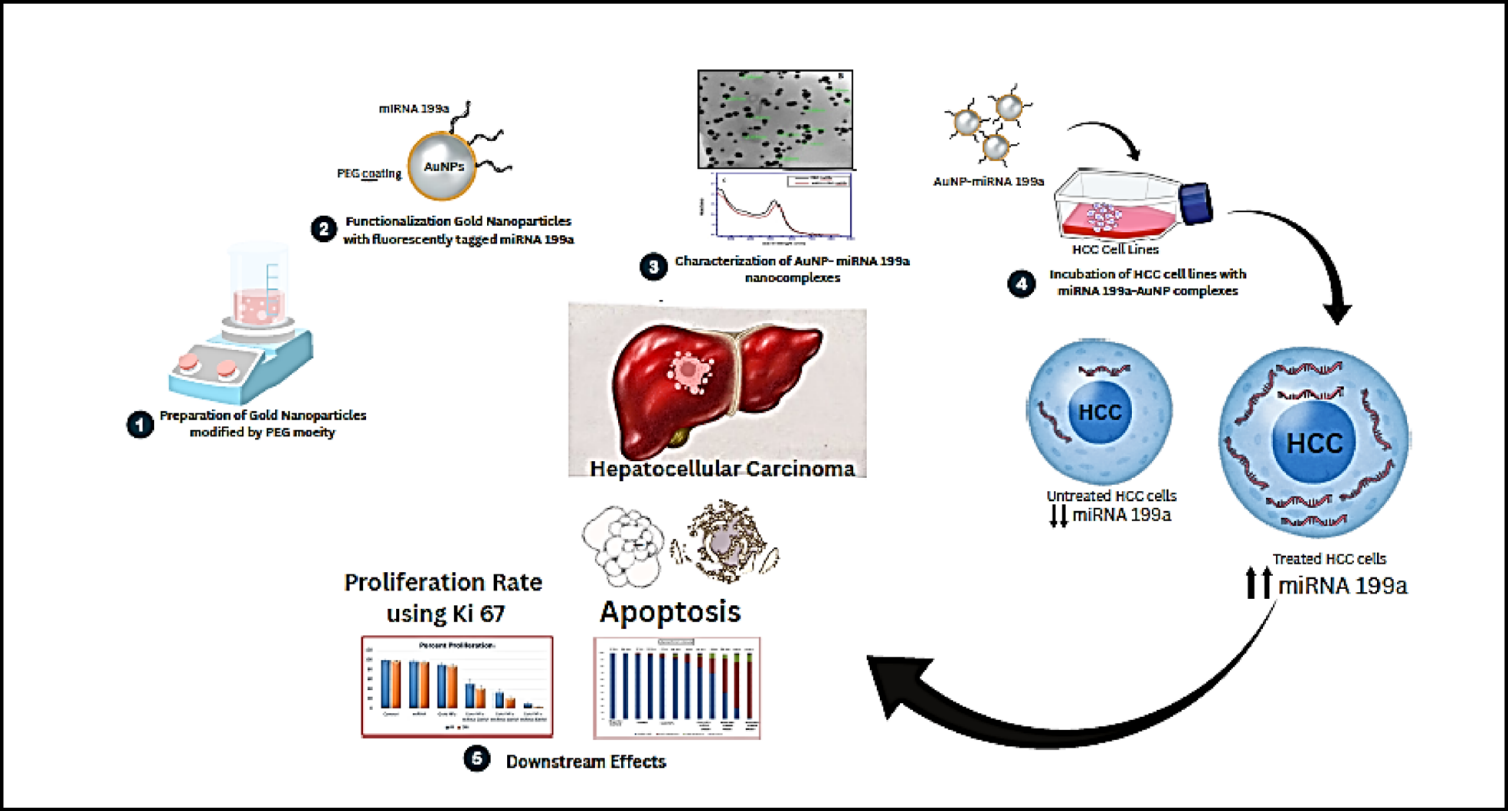
Schematic representation of the study.

Functional groups such as thiol and amino groups can be easily attached to the Au NPs surface, and these chemically modified Au NPs have been used as vehicles of miRNA. Functional Au NPs attach with miRNA199a have the ability to penetrate HepG2 cell lines leading to increase the level of miRNA199a into HepG2 cells, results in cell death, most likely through the induction of apoptosis and reduction of cell proliferation (Fig. 12).

## Materials and methods

### Materials

Tetrachloroauric acid trihydrated HAuCl_4_.3H_2_O, (Sigma Aldrich, Germany), dihydrate trisodium citrate Na_3_C_6_H_5_O_7_.2H_2_O (Oxford lab Chem, India), diethylpyrocarbonate (DEPC), Polyethylene glycol (PEG average molecular weight (Mn-800) (Sigma Aldrich, Germany), Oligonucleotide 5′-ACAGUAGUCUGCACAUUGGUUA-Cy5- 3′ was purchased (Gene Tech agency, United States), Dulbecco’s Modified Eagle Basal Media with high glucose (DMEM), Fetal Bovine Serum (FBS), and Phosphate Buffered Saline (PBS) at pH 7.4. without calcium and magnesium, Penicillin/ Streptomycin, Trypsin/EDTA and Trypan blue solution, (lonza, Switzerland), Propidium Iodide (PI) (component no. 51-66211E), 10X Annexin V, Binding Buffer (component no. 51-66121E) and Annexin V FITC, Ki-67 FITC ( miltenyi biotech -Germany), 3-(4,5-dimethylthiazol-2-yl)-2,5-diphenyltetrazolium bromide (MTT) (Serva – Germany), dimethyl sulfoxide (DMSO) (fisher scientific- UK). MTT assay (Serva – Germany).

### Gold nanoparticle synthesis

Citrate-capped gold nanoparticles (Au NPs) of ~ 15 nm diameter were synthesized according to the method reported by Crew et al.^44^. Briefly, 45 mL of Tetrachloroauric acid (1 mM) was boiled with magnetic stirrer (Thermo Scientific -Velp) stirring, then 5 mL of freshly prepared trisodium citrate (38.8 mM) was added. The solution was left until light yellow became deep crimson. Diethylpyrocarbonate (DEPC, 0.1%) was added to citrate-capped gold nanoparticles and agitated for 12 h.

### Capping of Au NPs with PEG

DEPC treated Au NP solution was mixed with NH2- PEG-SH to prepare PEG capped Au NPs (PEG-Au NPs)^45^. Briefly, 16.8 µg of NH2- PEG-SH was added to the 1 mL of DEPC treated Au NPs solution (1.9322 mM), the mixture was agitated at room temperature for 2 h to assist for the exchange of the citrate molecules with PEG molecules. The solution was then centrifuged at 15,000 rpm for 60 min, at 4 °C, in the sets of 1 mL^46^. The supernatant was discard and the pellet of Au NPs was mixed with DEPC treated water to make a total volume of 1 ml, the mixture was stirred well for 2 h.

### Loading of miRNA-199a on PEG capped Au NPs

1 mL of PEG-Au NPs was incubated with 1000 nM of miRNA-199a and stirred at 25 °C in a Thermomixer comfort, eppendorf for 24 h to yield fully functionalized miRNA-PEG-Au NPs. The solution containing the formulated nanoparticles was centrifuged at 15,000 rpm for 30 min at 4 °C to remove unbound miRNA. The nanoparticles were subsequently re-suspended in DEPC-treated water.

### Characterization of miRNA-199a loaded Au NPs

The optical characteristics of PEG-Au NPs and miRNA-PEG-Au NPs were analyzed by measuring the absorption maxima in the wavelength ranging from 250 to 800 nm at room temperature using nanodrop spectrophotometer (Nano drop Denovex DS-11 FX+). The surface plasmonic resonance (SPR) of electrons on the NP surface displays distinctive peaks at specific wavelengths. The hydrodynamic size, poly-dispersibility and zeta potential (ζ) for Au NPs, PEG-Au NPs and miRNA-PEG-Au NPs were measured using a zetasizer (Malvern, UK). The analysis was performed in triplicate at a temperature of 25 C°. The physical size and shape of prepared PEG-AuNPs and miRNA-PEG-Au NPs were assessed using a transmission electron microscope (JEOL-100 CX, Japan).

### Determining the miRNA-199a loading efficiency

The loading efficiency of mi-RNA on PEG capped AuNPs, was calculated by determining the free miRNA concentration^47^. The loading efficiency of mi-RNA onto the PEG capped Au NPs, the free miRNA concentration was determined. PEG-capped Au NPs were incubated and stirred with 1000 nM of miRNA for a duration of 24 h. Following incubation, the nanoparticles underwent centrifugation at 15,000 rpm for 30 min at 4 °C to eliminate unbound miRNA. The concentration of unbound miRNA in the supernatant was quantified by measuring absorbance using a UV–Vis spectrophotometer at 260 nm. The miRNA loading was quantified as the ratio of miRNA loaded to the total initial amount of miRNA added. The amount of miRNA loaded was determined using calibration curves that plot concentration against absorbance, utilizing known standards of the miRNA.

### Cytotoxicity of miRNA–PEG–Au NPs

The cytotoxic effects of miRNA-PEG-Au NPs was evaluated against the human hepatocellular carcinoma cell line in vitro, HepG2 cells supplied from the American Type Culture Collection (ATCC). Cells were 90% confluently plated on 96-well plates. Cells were treated with different formulations [Au NPs (80 nM), mi-RNA (80 nM) and mi-RNA loaded PEG-Au NPs (10, 20, 40, 80, 100, 150 nM) parallel with untreated HepG2 cells which were used as a negative control in all experiments, cells were incubated for 24, 48 and 72 h. MTT was used to measure cell viability. The medium was decanted and washed with PBS after incubation. To generate formazan crystals, 100 µl of 0.5 mg/ml MTT reagent was applied to each well and incubated for 3 h. After dissolving formazan crystals in DMSO, the optical density (OD) was measured at 570 nm using a microplate ELISA reader (Infinite F50, TECAN). The following formula was used to calculate cell viability:

$$Cell\;viability\left( \% \right) = \frac{{OD\;value\;of\;samples}}{{OD\;value\;of\;Controls}} \times 100$$

The average OD (± SD) was calculated, and the cytotoxicity curve was produced to estimate IC50 values. A cytotoxicity curve was plotted with the dose of the nanocomplex on the X-axis and the percentage cell viability on the Y-axis. The IC50 values corresponded to the dose at which 50% of the cells are viable, and 50% are dead.

### Apoptosis assay using annexin v flow cytometry^48^

Following product recommendations, the Annexin V FITC kit (Miltenyi Biotech, Auburn, CA, USA) was used for apoptosis testing. HepG2 cells were cultured in 6-well plates at a seeding density of 250,000 cells/well, cells were treated with different formulations [Au NPs (80 nM), mi-RNA (80 nM) and mi-RNA loaded PEG-AuNPs (20, 40, 80 nM] and incubated for 24 h. Cells were harvested and centrifuged. The cell pellet was resuspended in 1 ml of PBS and incubated with 0.25 µg/ml Annexin V reagent in 1X binding buffer for 15 min, followed by two washes with Wash Buffer. Cells were resuspended in binding buffer containing 0.5 µg/ml Propidium Iodide (PI). Component No. 51-66211E was incubated for 15 min in the dark at room temperature before being analyzed using a flow cytometer (Becton Dickinson, San Diego, CA, USA). Flow cytometry analysis was conducted utilizing a flow cytometer (BD, Bioscience). The evaluation of the data was conducted utilizing FACS Diva software (BD, Bioscience).

### Ki-67 staining protocol by flow cytometry^49^

To determine the effect of mi-RNA loaded PEG-Au NPs on the proliferative status of the HepG-2 cultured cells, proliferation marker (Ki-67) was quantitatively assessed using flowcytometry. Cells were plated in 6-well plates at a density of 250,000 cells per well and subsequently incubated. with the different formulations [Au NPs (80 nM), mi-RNA (80 nM) and mi-RNA loaded PEG-Au NPs (20, 40, 80 nM] for 24 h. Cells were harvested and centrifuged, the obtained pellet was mixed with 5 ml cold 70% − 80% ethanol, incubated at -20 °C for 2 h, washed with wash buffer three times. Then 20 µl of diluted Ki-67 was added according to the manufacturer’s instruction and mixed gently. The tubes were incubated at room temperature (RT) for 20–30 min in the dark. 2 ml of PBS washing buffer was added into each tube. Flow cytometry analysis was done utilizing a flow cytometer (BD, Bioscience). The data was analyzed utilizing FACS Diva software (BD, Bioscience).

### In vitro cellular uptake of the nanoparticles

#### Cellular uptake using confocal microscope^50^

The fluorescent images of HepG-2 were evaluated for the detection and localization of miRNA conjugated with Cy5 with confocal microscope at two-time intervals (4 and 24 h). HepG2 cells were plated on glass cover-slips with an initial cell density 1 × 10^5^ cell/ml and incubated with different formulations [AuNPs (80 nM), mi-RNA (80 nM) and mi-RNA loaded PEG-Au NPs (20, 40, 80 nM] parallel with untreated control cells for 4 h and 24 h. The cells underwent washing with PBS and were subsequently fixed with 4% paraformaldehyde. Permeabilization was conducted with 0.2% Triton X, followed by a 10-minute counterstaining with the nuclear stain Hoechst. Subsequently, the coverslips were mounted onto glass slides. Confocal laser scanning microscopy was conducted using a Leica TCS SPE II/DMi 8, accompanied by Leica LASX imaging software. The fluorescence intensity was analyzed using Image J (version 1.52p, NIH). The fluorescence intensity was calculated using the following equation^51^:

$$\begin{gathered} {\mathrm{Corrected}}\;{\mathrm{total}}\;{\mathrm{cell}}\;{\mathrm{fluorescence}} = {\text{Integrated density}} \hfill \\ \quad - \left( {{\mathrm{Area}}\;{\mathrm{of}}\;{\mathrm{selected}}\;{\mathrm{cell}} \times {\mathrm{Mean}}\;{\mathrm{fluorescence}}\;{\mathrm{of}}\;{\mathrm{background}}} \right) \hfill \\ \end{gathered}$$

#### Cellular uptake using transmission electron microscope

Cellular uptake of the mi-RNA loaded PEG-AuNPs at different concentrations (10, 40 and 80 nM) after incubation for 4, 24 h was assessed using TEM. Cells in each group were harvested by trypsinization and the resulting pellets from each group were processed to be examined by TEM (Jeol 100 CX, Japan). Examination of the sections was performed to assess the localization of the nanoparticles, as well as the cellular morphology.

## Data Availability

All data generated or analyzed during this study are included in this published article.

## Abbreviations

Au NPs: Gold nanoparticles
DEPC: Dimethylpyrocarbonate
DLS: Dynamic light scattering
DMEM: Dulbecco’s Modified Eagle’s Medium
DMSO: Dimethyl sulfoxide
HCC: Hepatocellular carcinoma
HepG2: Hepatocellular carcinoma cell line G2
HCV: Hepatitis C virus
HBV: Hepatitis B virus
IC50: Half-maximal inhibitory concentration
miRNA: MicroRNA
MMP-9: Matrix metalloproteinase 9
MTT: 3-(4,5-dimethythiazol-2-yl)-2,5-diphenyltetrazolium bromide
PDI: Poly dispersity index
PEG: Polyethylene glycol
PI: Propidium Iodide
SPR: Surface plasmonic resonance
TEM: Transmission electron microscope

## References

[1] Bray, F. et al. Global cancer statistics 2022: GLOBOCAN estimates of incidence and mortality worldwide for 36 cancers in 185 countries. *CA Cancer J. Clin.* **74**(3), 229–263 (2024). 38572751 10.3322/caac.21834

[2] Omar, A. et al. Egyptian society of liver cancer recommendation guidelines for the management of hepatocellular carcinoma. *J. Hepatocell Carcinoma* **10**, 1547–1571 (2023). 37744303 10.2147/JHC.S404424 PMC10516190

[3] Gomes, M. A., Priolli, D. G., Tralhao, J. G. & Botelho, M. F. Hepatocellular carcinoma: Epidemiology, biology, diagnosis, and therapies. *Rev. Assoc. Med. Bras.* **59**(5), 514– 424 (2013). 10.1016/j.ramb.2013.03.005 24041910

[4] Singal, A. G. et al. HCC surveillance improves early detection, curative treatment receipt, and survival in patients with cirrhosis: A meta-analysis. *J. Hepatol.* **77**(1), 128–139 (2022). 35139400 10.1016/j.jhep.2022.01.023 PMC9232881

[5] Djuranovic, S., Nahvi, A. & Green, R. A parsimonious model for gene regulation by miRNAs. *Science* **331**(6017), 550–553 (2011). 21292970 10.1126/science.1191138 PMC3955125

[6] Naidu, S., Magee, P. & Garofalo, M. MiRNA-based therapeutic intervention of cancer. *J. Hematol. Oncol.* **8**, 68 (2015). 26062952 10.1186/s13045-015-0162-0 PMC4465004

[7] Oura, K., Morishita, A. & Masaki, T. Molecular and functional roles of MicroRNAs in the progression of hepatocellular carcinoma-a review. *Int. J. Mol. Sci.* **21**, 8362 (2020). 10.3390/ijms21218362 PMC7664704 33171811

[8] Hu, Y. et al. MiR-199a-5p loss up-regulated DDR1 aggravated colorectal cancer by activating epithelial-to-mesenchymal transition related signaling. *Dig. Dis. Sci.* **59**(9), 2163–2172 (2014). 24711074 10.1007/s10620-014-3136-0

[9] Xu, X. et al. The Role of MicroRNAs in Hepatocellular Carcinoma. *J. Cancer* **9**(19), 3557–3569 (2018). 30310513 10.7150/jca.26350 PMC6171016

[10] Sohail, S. K. Natural products as modulators of miRNA in hepatocellular carcinoma: A therapeutic perspective. *J. Gene Med.* **27**(5), e70019 (2025). 40296860 10.1002/jgm.70019

[11] Jia, X. Q. et al. Lentivirus-mediated overexpression of microRNA-199a inhibits cell proliferation of human hepatocellular carcinoma. *Cell. Biochem. Biophys.* **62**(1), 237–244 (2012). 21847633 10.1007/s12013-011-9263-8

[12] Kim, J. H. et al. Anti-invasion and anti-migration effects of miR-199a-3p in hepatocellular carcinoma are due in part to targeting CD151. *Int. J. Oncol.* **49**(5), 2037–2045 (2016). 27599545 10.3892/ijo.2016.3677

[13] Li, B. et al. Mutual regulation of MiR-199a-5p and HIF-1alpha modulates the warburg effect in hepatocellular carcinoma. *J. Cancer* **8**(6), 940–949 (2017). 28529605 10.7150/jca.17496 PMC5436245

[14] Mao, B. & Wang, G. MicroRNAs involved with hepatocellular carcinoma (Review). *Oncol. Rep.* **34**(6), 2811–2820 (2015). 26398882 10.3892/or.2015.4275

[15] Shi, K. Q. et al. Hepatocellular carcinoma associated microRNA expression signature: Integrated bioinformatics analysis, experimental validation and clinical significance. *Oncotarget* **6**(28), 25093–25108 (2015). 26231037 10.18632/oncotarget.4437 PMC4694817

[16] Zhan, Y. et al. MiR-199a/b-5p inhibits hepatocellular carcinoma progression by post-transcriptionally suppressing ROCK1. *Oncotarget* **8**(40), 67169–67180 (2017). 28978024 10.18632/oncotarget.18052 PMC5620164

[17] Ghosh, A. et al. MiRNA199a-3p suppresses tumor growth, migration, invasion and angiogenesis in hepatocellular carcinoma by targeting VEGFA, VEGFR1, VEGFR2, HGF and MMP2. *Cell. Death Dis.* **8**(3), e2706 (2017). 28358369 10.1038/cddis.2017.123 PMC5386529

[18] Lee, J. M., Heo, M. J., Lee, C. G., Yang, Y. M. & Kim, S. G. Increase of miR-199a-5p by protoporphyrin IX, a photocatalyzer, directly inhibits E2F3, sensitizing mesenchymal tumor cells to anti-cancer agents. *Oncotarget* **6**(6), 3918–3931 (2015). 25714015 10.18632/oncotarget.2928 PMC4414163

[19] Xu, N. et al. Cisplatin-induced downregulation of miR-199a-5p increases drug resistance by activating autophagy in HCC cell. *Biochem. Biophys. Res. Commun.* **423**(4), 826–831 (2012). 22713463 10.1016/j.bbrc.2012.06.048

[20] Song, J. et al. MiR-199a regulates cell proliferation and survival by targeting FZD7. *PLoS One* **9**(10), e110074 (2014). 25313882 10.1371/journal.pone.0110074 PMC4196968

[21] Wang, H. A Review of nanotechnology in microRNA detection and drug delivery. *Cells* **13**(15), 1277 (2024). 10.3390/cells13151277 PMC11311607 39120308

[22] Moraes, F. C., Pichon, C., Letourneur, D. & Chaubet, F. miRNA delivery by nanosystems: State of the art and perspectives. *Pharmaceutics* **13**(11), 1901 (2021). 10.3390/pharmaceutics13111901 PMC8619868 34834316

[23] Chaudhary, V., Jangra, S. & Yadav, N. R. Nanotechnology based approaches for detection and delivery of microRNA in healthcare and crop protection. *J. Nanobiotechnol.* **16**(1), 40 (2018). 10.1186/s12951-018-0368-8 PMC5897953 29653577

[24] Fu, Y., Chen, J. & Huang, Z. Recent progress in microRNA-based delivery systems for the treatment of human disease. *ExRNA* **1**(1), 1–4 (2019).34171007

[25] Sousa, D. P. & Conde, J. Gold Nanoconjugates for miRNA modulation in cancer therapy: From miRNA silencing to miRNA mimics. *ACS Mater. Au* **2**(6), 626–640 (2022). 36397876 10.1021/acsmaterialsau.2c00042 PMC9650716

[26] Labatut, A. E. & Mattheolabakis, G. Non-viral based miR delivery and recent developments. *Eur. J. Pharm. Biopharm.* **128**, 82–90 (2018). 29679644 10.1016/j.ejpb.2018.04.018 PMC5984722

[27] Chen, Y., Xianyu, Y. & Jiang, X. Surface modification of gold nanoparticles with small molecules for biochemical analysis. *Acc. Chem. Res.* **50**(2), 310–319 (2017). 28068053 10.1021/acs.accounts.6b00506

[28] Jia, C. et al. Gold nanoparticle-based miR155 antagonist macrophage delivery restores the cardiac function in ovariectomized diabetic mouse model. *Int. J. Nanomed.* **12**, 4963–4979 (2017). 10.2147/IJN.S138400 PMC5513843 28744126

[29] Pourali, P. et al. Fate of the capping agent of biologically produced gold nanoparticles and adsorption of enzymes onto their surface. *Sci. Rep.* **13**(1), 4916 (2023). 36966192 10.1038/s41598-023-31792-5 PMC10039949

[30] Ghosh, R., Singh, L. C., Shohet, J. M. & Gunaratne, P. H. A gold nanoparticle platform for the delivery of functional microRNAs into cancer cells. *Biomaterials* **1**(3), 807–816 (2013). 10.1016/j.biomaterials.2012.10.023 23111335

[31] Giljohann, D. A., Seferos, D. S., Prigodich, A. E., Patel, P. C. & Mirkin, C. A. Gene regulation with polyvalent siRNA-nanoparticle conjugates. *J. Am. Chem. Soc.* **131**(6), 2072–2073 (2009). 19170493 10.1021/ja808719p PMC2843496

[32] Bahadur, K. C. R., Thapa, B. & Bhattarai, N. Gold nanoparticle-based gene delivery: Promises and challenges. *Nanatechnol. Rev.* **1**(3), 269–280 (2014).

[33] Lee, S. H., Bae, K. H., Kim, S. H., Lee, K. R. & Park, T. G. Amine-functionalized gold nanoparticles as non-cytotoxic and efficient intracellular siRNA delivery carriers. *Int. J. Pharm.* **19**(1), 94–101 (2008). 10.1016/j.ijpharm.2008.07.027 18723087

[34] Shipway, A. N., Katz, E. & Willner, I. Nanoparticle arrays on surfaces for electronic, optical, and sensor applications. *ChemPhysChem* **4**(1), 18–52 (2000). 10.1002/1439-7641(20000804)1:1<18::AID-CPHC18>3.0.CO;2-L 23696260

[35] Cai, H. et al. Gold nanoparticles-loaded anti-miR221 enhances antitumor effect of sorafenib in hepatocellular carcinoma cells. *Int. J. Med. Sci.* **16**(12), 1541–1548 (2019). 31839741 10.7150/ijms.37427 PMC6909811

[36] Wang, H. et al. Ultrasound-guided microbubble-mediated locoregional delivery of multiple MicroRNAs improves chemotherapy in hepatocellular carcinoma. *Nanotheranostics* **6**(1), 62–78 (2022). 34976581 10.7150/ntno.63320 PMC8671967

[37] El-Naggar, N. E. et al. Process optimization for gold nanoparticles biosynthesis by *Streptomyces albogriseolus* using artificial neural network, characterization and antitumor activities. *Sci. Rep.* **14**(1), 4581 (2024). 38403677 10.1038/s41598-024-54698-2 PMC10894868

[38] Chen, Y., Gao, D. Y. & Huang, L. In vivo delivery of miRNAs for cancer therapy: Challenges and strategies. *Adv. Drug Deliv. Rev.* **81**, 128–141 (2015). 24859533 10.1016/j.addr.2014.05.009 PMC5009470

[39] Hu, Y. et al. Exosomes derived from bone mesenchymal stem cells alleviate compression-induced nucleus pulposus cell apoptosis by inhibiting oxidative stress. *Oxid. Med. Cell. Longev.* **2021**, 2310025 (2021). 34733401 10.1155/2021/2310025 PMC8560283

[40] Mostafa, S. M., Gamal-Eldeen, A. M., Maksoud, N. A. E. & Fahmi, A. A. Epigallocatechin gallate-capped gold nanoparticles enhanced the tumor suppressors let-7a and miR-34a in hepatocellular carcinoma cells. *Acad. Bras. Cienc.* **92**(4), e20200574 (2020). 10.1590/0001-3765202020200574 33206791

[41] Baghani, L. et al. Trimethyl-chitosan coated gold nanoparticles enhance delivery, cellular uptake and gene silencing effect of EGFR-siRNA in breast cancer cells. *Front. Mol. Biosci.* **9**, 871541 (2022). 35517864 10.3389/fmolb.2022.871541 PMC9065351

[42] Ren, K. L. T., Zhang, W., Ren, J., Li, Z. & Wu, G. miR-199a-3p inhibits cell proliferation and induces apoptosis by targeting YAP1, suppressing Jagged1-Notch signaling in human hepatocellular carcinoma. *J. Biomed. Sci.* **10**(1), 79 (2016). 10.1186/s12929-016-0295-7 PMC5103406 27832779

[43] Mahati, S., Fu, X., Ma, X., Zhang, H. & Xiao, L. Delivery of miR-26a using an exosomes-based nanosystem inhibited proliferation of hepatocellular carcinoma. *Front. Mol. Biosci.* **8**, 738219 (2021). 34552961 10.3389/fmolb.2021.738219 PMC8450326

[44] Crew, E. et al. MicroRNA conjugated gold nanoparticles and cell transfection. *Anal. Chem.* **84**(1), 26–29 (2012). 22148593 10.1021/ac202749p

[45] Manson, J. K. D., Meenan, B. J. & Dixon, D. Polyethylene glycol functionalized gold nanoparticles: The influence of capping density on stability in various media. *Gold Bull.* **44**(2), 99–105 (2011).

[46] Kim, J. H. et al. Effective delivery of anti-miRNA DNA oligonucleotides by functionalized gold nanoparticles. *J. Biotechnol.* **155**(3), 287–292 (2011). 21807040 10.1016/j.jbiotec.2011.07.014

[47] Jensen, S. A. et al. Spherical nucleic acid nanoparticle conjugates as an RNAi-based therapy for glioblastoma. *Sci. Transl. Med.* **30**(209), 209ra152 (2013). 10.1126/scitranslmed.3006839 PMC4017940 24174328

[48] van Engeland, M., Ramaekers, F. C., Schutte, B. & Reutelingsperger, C. P. A novel assay to measure loss of plasma membrane asymmetry during apoptosis of adherent cells in culture. *Cytometry* **24**(2), 131–139 (1996). 8725662 10.1002/(SICI)1097-0320(19960601)24:2<131::AID-CYTO5>3.0.CO;2-M

[49] Kubbutat, M. H. et al. Epitope analysis of antibodies recognising the cell proliferation associated nuclear antigen previously defined by the antibody Ki-67 (Ki-67 protein). *J. Clin. Pathol.* **47**(6), 524–528 (1994). 7520455 10.1136/jcp.47.6.524 PMC494740

[50] Cogswell, C. J. Confocal differential interference contrast (DIC) microscopy: Including a theoretical analysis of conventional and confocal DIC imaging. *J. Microsc.* **165**(1), 81–101 (1992).

[51] Awaad, A. K. et al. The role of hepatic transcription factor cAMP response element-binding protein (CREB) during the development of experimental nonalcoholic fatty liver: A biochemical and histomorphometric study. *Egypt. Liver J.* **28**(1), 36 (2020).

