# Correction: Anticarcinogenic effects of miR-199a-loaded gold nanoparticles on hepatocellular carcinoma: in vitro study

**DOI:** 10.1038/s41598-026-57367-8

**Published:** 2026-06-16

**Authors:** Samar El Achy, Maisa E. Moustafa, Mohamed Fouad, Ashraf Awad, Reham Abdelhaleem, Thanaa Shalaby

**Affiliations:** 1 Department of Pathology, Faculty of Medicine, Alexandria University, Alexandria, 21521 Egypt; 2 Center of Excellence for Research in Regenerative Medicine and Applications (CERRMA), Faculty of Medicine, Alexandria University, Alexandria, 21521 Egypt; 3 Department of Medical Biophysics, Medical Research Institute, Alexandria University, Alexandria, 21521 Egypt; 4 Department of Biochemistry, Medical Research Institute, Alexandria University, Alexandria, 21521 Egypt; 5 Department of Clinical Pathology, Faculty of Medicine, Alexandria University, Alexandria, 21521 Egypt; 6 Nanotechnology Training Center, Medical Technology Center, Alexandria University, Alexandria, Egypt

Correction to: *Scientific Reports* 10.1038/s41598-026-42604-x, published online 02 April 2026

The original version of this Article contained an error in Figure 7, where the confocal microscopy images in panel A were a duplication of those in panel C. The original Figure 7 and accompanying legend appear below.

**Fig. 7:**
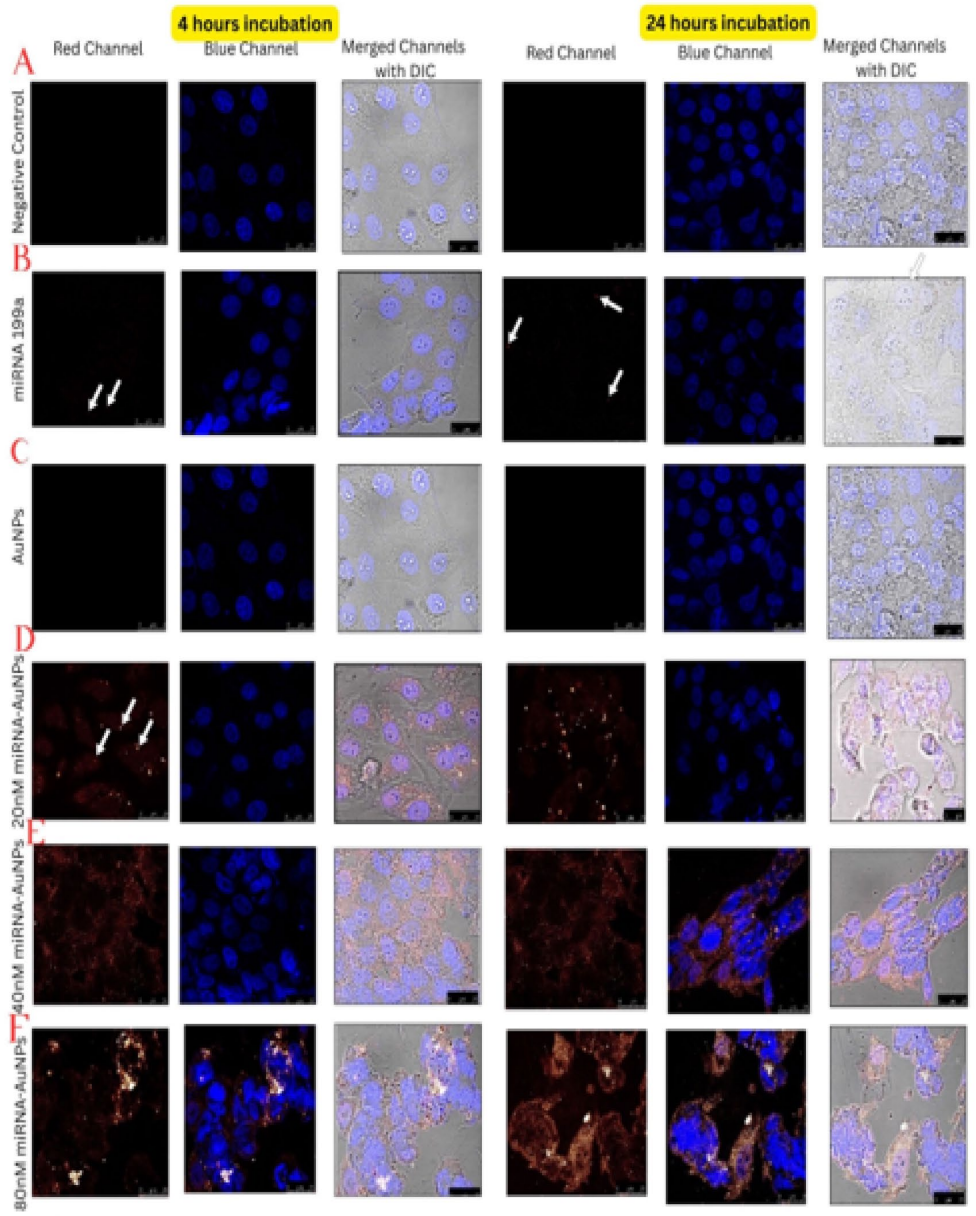
Confocal Laser Scanning Photomicrograph of negative control HepG-2 cells (first row), HepG-2 cells treated with miRNA/Cy5 (2nd row), HepG-2 cells treated with gold nanoparticles (3rd row), HepG-2 cells treated with 20 nM miRNA–PEG–AuNPs (4th row), HepG-2 cells treated with 40 nM miRNA–PEG–AuNPs (5th row), HepG-2 cells treated with 80 nM miRNA–PEG–AuNPs (6th row) after 4 and 24 h incubation time. The blue fluorescence represents the nuclear counterstaining with Hoechst stain, the negative control cells showed no red fluorescence (black background). DIC mode showing the epithelial-like morphology of the HepG2 cells in small aggregates. Merged image from blue channel and red channel.

The original Article has been corrected.

